# Comprehensive Analysis of IGFBPs as Biomarkers in Gastric Cancer

**DOI:** 10.3389/fonc.2021.723131

**Published:** 2021-10-21

**Authors:** Qi Liu, Jianwu Jiang, Xiefu Zhang, Meixiang Zhang, Yang Fu

**Affiliations:** ^1^Department of Gastrointestinal Surgery, The First Affiliated Hospital of Zhengzhou University, Zhengzhou, China; ^2^Center for Reproductive Medicine, Henan Key Laboratory of Reproduction and Genetics, The First Affiliated Hospital of Zhengzhou University, Zhengzhou, China

**Keywords:** gastric cancer, insulin-like growth factor-binding protein, prognostic biomarker, bioinformatics analysis, stomach adenocarcinoma (STAD)

## Abstract

**Objective:**

Gastric cancer is the fifth most common cancer worldwide and the third leading cause of cancer-related deaths. Insulin-like growth-factor-binding proteins (IGFBPs) were initially identified as passive inhibitors that combined with insulin-like growth factors (IGFs) in serum. However, more recent data have shown that they have different expression patterns and a variety of functions in the development and occurrence of cancers. Thus, their various roles in cancer still need to be elucidated. This study aimed to explore the IGFBPs and their prognostic value as markers in gastric cancer.

**Methods:**

Oncomine, Gene Expression Profiling Interactive Analysis (GEPIA), Kaplan–Meier Plotter, cBioPortal, GeneMANIA, and TIMER were used to analyze the differential expression, prognostic value, genetic alteration, and association with immune cell infiltration of IGFPBs in gastric cancer.

**Results:**

Expression levels of IGFBP3, IGFBP4, and IGFBP7 were significantly elevated in gastric cancer tissues, whereas those of IGFBP1 were reduced in normal tissues. IGFBP1/5/7 expression was significantly associated with overall survival whereas IGFBP6/7 expression was significantly correlated with disease-free survival in gastric cancer patients. IGFBP3/5/6/7 were associated with clinical cancer stage. Gene ontology and Kyoto Encyclopedia of Genes and Genome analyses showed that IGFBP3/5/7 were mainly enriched in focal adhesion, extracellular matrix structural constituent, cell-substratist junction, extracellular structure, and matrix organization. Stomach adenocarcinoma (STAD) and gastric cancer had more IGFBP1–7 mutations than other tumor types. Hub gene analysis showed that TP53 and IGF2 expression was significantly elevated in STAD patients; PLG, PAPPA, AFP, and CYR61 were associated with overall survival rate; and IGFALS, PLG, IGF1, AHSG, and FN1 were associated with disease-free survival. Finally, IGFBP3–7 were all associated with cancer-associated fibroblast infiltration in STAD, colon adenocarcinoma, and rectal adenocarcinoma.

**Conclusion:**

Our study provides a comprehensive analysis and selection of IGFBPs as prognostic biomarkers in STAD. This was the first bioinformatic analysis study to describe the involvement of IGFBPs, especially IGFBP7, in gastric cancer development through the extracellular matrix.

## Introduction

Stomach cancer is the fifth most common cancer worldwide (1). More than 900,000 gastric cancer cases are diagnosed each year, with higher incidences among males and in developing countries (2). Most gastric cancers are already at an advanced stage when they are diagnosed; thus, gastric cancer has become the third leading cause of cancer-related deaths, causing 784,000 deaths globally in 2018 (3). Ninety percent of gastric cancers are gastric adenocarcinomas in terms of pathological type. However, their biological behaviors and histopathological structures vary, as do patients’ outcomes.

The pathogenesis of gastric cancer is unclear; infections, genetic mutations, and unhealthy lifestyles are the main causes. *Helicobacter pylori* infection is the best-described risk factor for non-cardia gastric cancer. Chronic *H. pylori* infection leads to atrophic gastritis and intestinal metaplasia, which are considered to be precancerous lesions (4). Familial aggregation appears in approximately 10% of all gastric cancer cases, and germline mutations are found in 1%–3% of gastric cancer patients (5). For instance, a pathogenic gene in STAD, E­cadherin-coding gene CDH1, appears in 30%–40% of hereditary diffuse gastric cancer patients (6). CTNNA1, a cell matrix αE­catenin-coding gene, has an exon 1B point mutation also found in families with hereditary diffuse gastric cancer (7, 8). APC, a tumor suppressor and Wnt signaling pathway antagonist-coding gene, also plays a part in gastric adenocarcinoma by altering cell migration and adhesion (9). These two genes indicate the importance of the tumor microenvironment (TME), which contains multiple cell types that enable the sustained growth, invasion, and metastasis of cancers. With respect to lifestyle, cigarette smoking, alcohol consumption, salty food intake, and older age are risk factors for gastric cancer, whereas a high intake of vegetables and fruit and a low-salt diet will reduce the risk (10).

Insulin-like growth-factor-binding proteins IGFBPs are a series of cystine-rich proteins that act as combiners of insulin growth factors (IGFs) in serum. They have important roles in tumor occurrence and development, prolonging the half-life of the IGFs, controlling their access to IGF receptors (IGFRs), and promoting or inhibiting IGF downstream signaling pathways (11). Recent studies have indicated that these growth factors are also involved in interaction with ECM proteins and proteolytic enzymes (12). This regulation process is also called the IGF–IGFR–IGFBP axis. IGFBPs can be divided into two groups according to their different affinities for IGFs: high-affinity binding proteins (IGFBP1–6) and low-affinity binding proteins (IGFBP7–10). Our study focused on the prognostic value of IGFBP1–6 in gastric cancer; however, IGFBP7 is also significantly upregulated in STAD patients and closely related to prognosis (13). Thus, we also included IGFBP7 in the analysis.

## Materials and Methods

### Oncomine

IGFBP1–7 mRNA levels in diverse cancer types were analyzed using Oncomine (www.oncomine.org), which provides microarray information for 65 gene expression datasets comprising most major cancer types (14). In this study, a p-value <0.01, a fold change of 2, and a gene rank in the top 10% were set as the significance thresholds. Student’s t-test was applied to determine the differences in expression of IGFBP1–7 in gastric cancer.

### Gene Expression Profiling Interactive Analysis

Gene Expression Profiling Interactive Analysis (GEPIA) (http://gepia.cancer-pku.cn) is an interactive web server using a standard procession pipeline to analyze 9,736 tumor tissues and 8,587 normal samples from The Cancer Genome Atlas (TCGA) and the GTEx project (15). In this study, IGFBP1–7 expression in normal and tumor tissues was compared by Student’s t-test. IGFBP1–7 expression between different stages was compared with one-way analysis of variance. Survival analysis was performed with Kaplan–Meier curves. Comparisons of normal and tumor tissues and survival analysis were also performed for hub genes of IGFBP1–7 by GEPIA.

### cBioPortal

cBioPortal (www.cbioportal.org) is a comprehensive cancer data analysis tool. It provides online analysis of data types including gene mutation, copy number variation, mRNA expression, and protein phosphorylation (16). In this study, genetic alterations (structure variant, mutation, and copy number variant data) of IGFBP1–7 from 11,084 samples (from 11,070 patients in 35 studies) were obtained from cBioPortal.

### STRING

STRING (https://string-db.org/) is a database of protein–protein interactions (PPIs), which can be used to predict a comprehensive and global network for a customized protein list (17). In this study, IGFBP1–7 PPI network analysis was performed with STRING.

### TIMER

TIMER (https://cistrome.shinyapps.io/timer/) is a web resource that can be used to evaluate immune cell infiltration and its clinical effects (18). IGFBP1–7 immune cell infiltration levels in STAD were analyzed and visualized using scatterplots with TIMER.

### Other Bioinformatic Analyses

Gene expression data for STAD in HTSeq-FPKM format were downloaded from TCGA, and 407 patients were selected for analysis. The R package “pROC” was used for ROC analysis, and “ggplot2” was used for visualization. Genes co-expressed with IGFBP3/5/7 were screened from TCGA data with R package “stat” using Pearson correlation with coefficient |r| > 0.4 and p < 0.001. Gene ontology (GO) and Kyoto Encyclopedia of Genes and Genome (KEGG) analysis were performed on co-expressed genes with the R package “clusterProfiler” to explore possible biological functions and signaling pathways affected by IGFBP1–7. GO analysis included biological process, cell composition, and molecular function (p < 0.05 was considered to indicate statistical significance) (19).

## Results

### Differential Expression of IGFBP1–7 in Gastric Cancer

**Figure 1:**
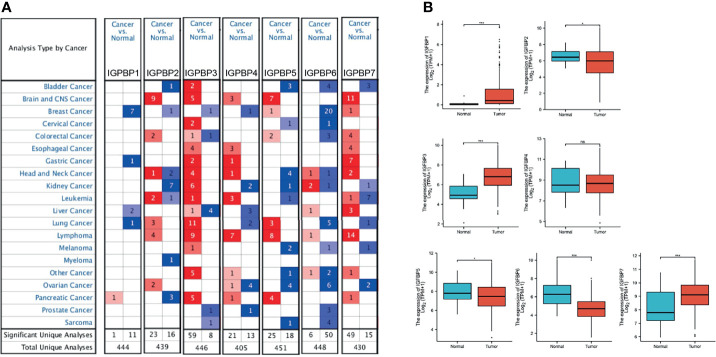
IGFBP1–7 expression in STAD patients. **(A)** mRNA expression of IGFBP1–7 in different cancer types from Oncomine. The graphic shows the numbers of datasets with statistically significant alterations in the mRNA expression of the target gene: upregulated (red) and downregulated (blue). The following criteria were used: p-value: 0.01, fold change: 2, gene rank: 10%, data type: mRNA, analysis type: cancer *vs.* normal tissue. As shown in the green frame, transcriptional levels of IGFBP3/4/7 were significantly elevated while transcriptional levels of IGFBP1 were reduced in gastric cancer. **(B)** Expression of IGFBP1–7 in the TCGA database. *p < 0.05, ***p < 0.001.

IGFBP1–7 expression data were analyzed in the Oncomine database. Expression of IGFBP3/4/7 was significantly elevated in gastric cancer samples, whereas IGFBP1 expression was decreased in normal tissues. Specific fold change and p-values are listed in **Table 1**. Based on the Oncomine data, the following expression fold change values relative to the corresponding normal tissues were obtained: 4.577 (p = 9.92E-09) for IGFBP3 in gastric mixed adenocarcinoma; 3.73 (p = 6.31E-06) for IGFBP4 in gastric cancer; 4.217 (p = 6.31E-13) for IGFBP7 in diffuse gastric adenocarcinoma; 2.333 (p = 6.19E-19) for gastric intestinal type adenocarcinoma; 4.141 (p = 1.24E-05) for gastric mixed adenocarcinoma (20); and 2.926 (p = 7.51E-06) for gastric mixed adenocarcinoma (21). That is, IGFBP3, IGFBP4, and IGFBP7 expression levels were higher in gastric cancer patients *vs.* normal in the Oncomine data (**Figure 1A**). In TCGA data, the average IGFBP1/3/7 expression levels in STAD were significantly higher than those in normal tissue, while IGFBP2/5/6 expression was significantly lower in tumor tissue (**Figure 1B**). Analysis of TCGA STAD data showed that 4%–6% of STAD patients had high expression of IGFBP1–7. These data suggest that IGFBP1/3/7 might have key roles in gastric cancer.

**Table 1:** IGFBP1–7 expression in STAD patients from the cBioPortal database.

	Type of gastric cancer *versus* normal gastric tissue	Fold change	p value	t test	Source and/or reference	
IGFBP1	Gastric intestinal type adenocarcinoma *vs.* normal	1.068	2.96E-08	6.685	Deng Gastric	PMID: 22315472
	Diffuse gastric adenocarcinoma *vs.* normal	1.033	1.02E-05	4.845	Deng Gastric	PMID: 22315472
	Gastric mixed adenocarcinoma *vs.* normal	1.051	0.009	2.807	Deng Gastric	PMID: 22315472
	Gastric cancer *vs.* normal	1.057	9.27E-05	4.099	Deng Gastric	PMID: 22315472
	Gastric adenocarcinoma *vs.* normal	1.045	3.01E-05	4.293	Deng Gastric	PMID: 22315472
	Gastric intestinal type adenocarcinoma *vs.* normal	1.13	4.04E-08	6.545	TCGA	
	Mucinous gastric adenocarcinoma *vs.* normal	1.078	0.002	3.518	TCGA	
	Diffuse gastric adenocarcinoma *vs.* normal	1.078	9.35E-06	4.798	TCGA	
	Gastric tubular adenocarcinoma *vs.* normal	1.123	6.52E-05	4.387	TCGA	
	Gastric adenocarcinoma *vs.* normal	1.101	1.88E-15	8.638	TCGA	
IGFBP2	Gastric adenocarcinoma *vs.* normal	1.017	0.001	3.172	Deng Gastric	PMID: 22315472
IGFBP3	Gastric mixed adenocarcinoma *vs.* normal	4.577	9.92E-09	11.971	Chen Gastric	PMID: 12925757
	Gastric intestinal type adenocarcinoma *vs.* normal	2.319	7.89E-11	7.202	Chen Gastric	PMID: 12925758
	Gastric intestinal type adenocarcinoma *vs.* normal	1.068	2.96E-08	6.685	Deng Gastric	PMID: 22315472
	Diffuse gastric adenocarcinoma *vs.* normal	1.033	1.02E-05	4.845	Deng Gastric	PMID: 22315472
	Gastric mixed adenocarcinoma *vs.* normal	1.051	0.009	2.807	Deng Gastric	PMID: 22315472
	Gastric cancer *vs.* normal	1.057	9.27E-05	4.099	Deng Gastric	PMID: 22315472
	Gastric adenocarcinoma *vs.* normal	1.045	3.01E-05	4.293	Deng Gastric	PMID: 22315472
	Gastric intestinal type adenocarcinoma *vs.* normal	1.13	4.04E-08	6.545	TCGA	
	Mucinous gastric adenocarcinoma *vs.* normal	1.078	0.002	3.518	TCGA	
	Diffuse gastric adenocarcinoma *vs.* normal	1.078	9.35E-06	4.798	TCGA	
	Gastric tubular adenocarcinoma *vs.* normal	1.123	6.52E-05	4.387	TCGA	
	Gastric adenocarcinoma *vs.* normal	1.101	1.88E-15	8.638	TCGA	
IGFBP4	Gastric cancer *vs.* normal	3.731	6.31E-06	5.498	Wang Gastric	PMID: 21132402
	Diffuse gastric adenocarcinoma *vs.* normal	1.84	2.01E-05	4.526	Cho Gastric	PMID: 21447720
	Gastric adenocarcinoma *vs.* normal	1.965	0.021	2.993	Cho Gastric	PMID: 21447720
	Gastric intestinal type adenocarcinoma *vs.* normal	1.73	0.01	2.483	Cho Gastric	PMID: 21447720
	Gastric mixed adenocarcinoma *vs.* normal	1.647	0.018	2.379	Cho Gastric	PMID: 21447720
IGFBP5	Diffuse gastric adenocarcinoma *vs.* normal	1.78	0.000557	3.678	Chen Gastric	PMID: 12925758
IGFBP6	NA					
IGFBP7	Diffuse gastric adenocarcinoma *vs.* normal	4.217	6.31E-13	14.986	Chen Gastric	PMID: 12925758
	Gastric intestinal type adenocarcinoma *vs.* normal	2.333	6.19E-19	11.245	Chen Gastric	PMID: 12925758
	Gastric mixed adenocarcinoma *vs.* normal	4.141	1.24E-05	8.377	Chen Gastric	PMID: 12925758
	Gastric cancer *vs.* normal	2.926	7.51E-06	5.352	Wang Gastric	PMID: 21132402
	Gastric mixed adenocarcinoma *vs.* normal	4.669	1.54E-06	7.154	DErrico Gastric	PMID: 19081245
	Gastric intestinal type adenocarcinoma *vs.* normal	2.721	3.26E-09	7.102	DErrico Gastric	PMID: 19081245
	Diffuse gastric adenocarcinoma *vs.* normal	2.238	4.16E-06	4.998	Cho Gastric	PMID: 21447720
	Gastric cancer *vs.* normal	1.466	0.000307	3.497	Cui Gastric	PMID: 20965966

### Prognostic Value of mRNA Expression of IGFBP1–7 in STAD Patients

**Figure 2:**
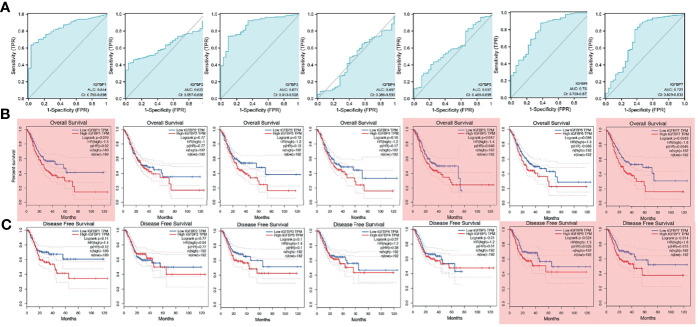
Survival analysis and diagnostic value of IGFBP1–7 in STAD patients. **(A)** Diagnostic value of IGFBP1–7 in STAD patients. **(B)** Overall survival curve for IGFBP1–7 in STAD patients. **(C)** Disease-free survival curve for IGFBP1–7 in STAD patients. Results with p < 0.05 are marked with red shadow.

To investigate the prognostic value of IGFBP1–7 in STAD patients, area under the curve (AUC) analysis was performed for IGFBP1–7 in TCGA STAD mRNA data. The respective AUCs for IGFBP1–7 were 0.844, 0.662, 0.871, 0.487, 0.597, 0.793, and 0.721 (**Figure 2A**). Kaplan–Meier Plotter was used for survival analysis with GEPIA; IGFBP1/5/7 mRNA levels were found to be significantly associated with overall survival (**Figure 2B**), whereas IGFBP6/7 mRNA levels were significantly correlated with disease-free survival rates (**Figure 2C**). All these data indicated significant roles of IGFBP1/5/6/7 in STAD. Prognostic value of IGFBPs were also validated in another cohort (20), but IGFBP1/3/5 expression was not significantly associated with overall survival. To investigate the relationships between IGFBP1–7 expression and clinicopathological parameters in STAD patients, we analyzed mRNA levels in patients of different gender, age, *H. pylori* infection status, metastasis, pathological stage, lymphatic metastasis, and T stage. The results showed that IGFBP1–7 expression was not related to patients’ age, gender, *H. pylori* infection status, metastasis, or lymphatic metastasis; however, IGFBP3/5/6/7 expression was significantly elevated in pathological stages II–IV compared with pathological stage I. IGFBP3/5/7 expression was also significantly increased in advanced T-stage patients. These results suggest that IGFBP3/5/6/7 may have roles in tumor progression (**Table 2**). Cox multivariate regression analysis showed that besides tumor stages, IGFBP1 and IGFBP7 were independent predictors in STAD patients (**Supplementary Table 1**).

**Table 2:** The relationships between IGFBP1/3/5/7 with clinicopathological parameters in STAD patients.

Clinicopathological parameters	N	IGFBP1 expression		IGFBP2 expression		IGFBP3 expression		IGFBP4 expression		IGFBP5 expression		IGFBP6 expression		IGFBP7 expression	
		Mean±SD	P value	Mean±SD	P value	Mean±SD	P value	Mean±SD	P value	Mean±SD	P value	Mean±SD	P value	Mean±SD	P value
Tissue			<0.001		0.022*		<0.001		0.803*		0.069		<0.001*		<0.001
Normal	32	0.095±0.162		6.539±0.727		5.052±1.010		8.791±1.246		7.918±1.253		6.383±1.602		8.138±1.302	
Tumor	375	1.071±1.402		5.762±1.872		6.787±1.199		8.695±1.286		7.407±1.415		4.706±1.216		9.128±1.048	
Gender			0.826*		0.691*		0.867		0.626		0.809		0.596*		0.845
Female	134	1.044±1.331		5.722±1.830		6.773±1.235		8.652±1.372		7.384±1.428		4.654±1.255		9.113±1.104	
Male	241	1.087±1.443		5.785±1.898		6.795±1.181		8.72±1.238		7.421±1.41		4.735±1.196		9.136±1.019	
Age(years)			0.107*		0.006*		0.509		0.294		0.166		<0.001*		0.107*
≤65	164	0.893±1.176		6.088±1.707		6.827±1.23		8.776±1.359		7.521±1.396		4.941±1.215		9.236±1.147	
>65	207	1.22±1.555		5.489±1.967		6.744±1.184		8.634±1.237		7.316±1.42		4.53±1.191		9.04±0.968	
H pylori infection			0.438*		0.123*		0.904		0.197		0.376		0.344		0.008
Yes	18	0.742±0.889		5.389±1.308		6.479±1.098		8.729±1.027		7.372±1.37		4.612±1.013		9.348±0.81	
No	145	1.139±1.442		5.866±1.864		6.517±1.274		8.34±1.221		7.045±1.486		4.345±1.134		8.701±0.983	
Metastasis			0.305*		0.134*		0.744		0.348		0.987		0.975		0.847*
No	330	1.039±1.415		5.725±1.889		6.763±1.203		8.66±1.292		7.398±1.425		4.71±1.237		9.117±1.043	
Yes	25	1.320±1.424		6.217±2.003		6.845±1.156		8.911±1.219		7.403±1.512		4.718±1.18		9.250±1.204	
Pathological stage			0.758*		0.414*		0.001		0.053		<0.001		0.002		<0.001
I	53	1.190±1.637		5.599±2.156		6.274±1.414		8.393±1.488		6.563±1.585		4.25±1.316		8.608±1.128	
II-IV	150	1.062±1.367		5.843±1.833		6.857±1.14		8.76±1.228		7.55±1.336		4.82±1.195		9.215±1.008	
Lymphatic metastasis			0.631*		0.775*		0.608*		0.844		0.055		0.993		0.613
No	111	1.094±1.580		5.732±2.064		6.769±1.274		8.722±1.376		7.197±1.475		4.716±1.368		9.086±1.176	
Yes	246	1.063±1.340		5.829±1.803		6.788±1.165		8.693±1.226		7.505±1.368		4.718±1.153		9.147±0.984	
T stage			0.958*		0.828*		0.015		0.043		<0.001		0.081		<0.001
T1-T2	99	1.105±1.540		5.839±1.982		6.523±1.337		8.465±1.355		6.963±1.507		4.496±1.258		8.799±1.064	
T3-T4	268	1.055±1.352		5.784±1.824		6.866±1.138		8.771±1.248		7.549±1.355		4.787±1.208		9.231±1.025	

IGF, Insulin-like growth factor-binding protein; STAD, Stomach adenocarcinoma.*samples do not meet the normal distribution, use Mann-Whitney U test.

### Correlations Between IGPBP1–7 Expression and Tumor Stage in STAD Patients

**Figure 3:**
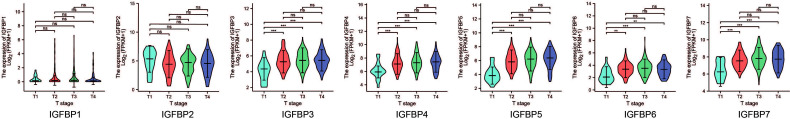
Difference between IGPBP1–7 expression and tumor stage in STAD patients from the TCGA database.

To further study the functions of IGFBP1–7 in tumor progression, we analyzed their expression levels at different stages using TCGA data. Violin plots of IGFBP1–7 expression at different tumor stages showed an upward trend with increasing T stages. This trend was significant for IGFBP3/4/5/6/7 (**Figure 3**). Based on these results, combined with those of the mRNA expression analysis, IGFBP3/5/7 were chosen for further study of the mechanisms of tumor progression.

### Analysis of Genes Co-Expressed With IGFBP3/5/7 in STAD Patients

**Figure 4:**
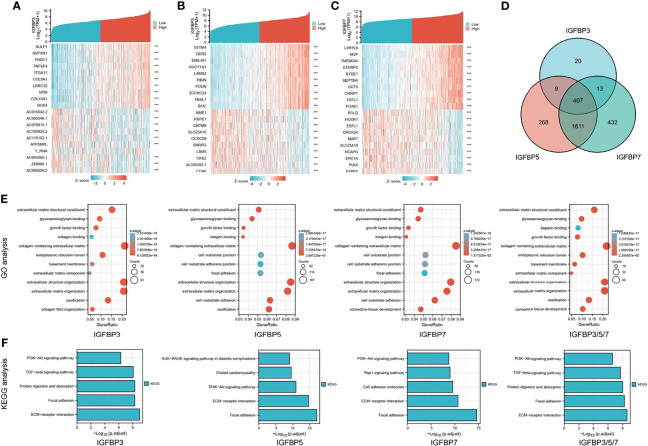
Heat map, Venn diagram, and GO/KEGG analysis of IGFBP3/5/7. Heat map and Venn diagram showing top 10 genes with positive and negative co-expression with IGFBP3 **(A)**, IGFBP5 **(B)**, and IGFBP7 **(C)** in STAD patients from the TCGA database. **(D)** Intersection of genes co-expressed with IGFBP3/5/7. |r| > 0.4, p < 0.001. **(E)** GO analysis of IGFBP3, IGFBP5, IGFBP7, and IGFBP3/5/7 (407 overlap genes); **(F)** KEGG analysis of IGFBP3, IGFBP5, IGFBP7, and IGFBP3/5/7 (407 overlap genes).

To further understand the possible molecular mechanisms of IGFBP3/5/7 in tumor progression, we selected the top 10 genes positively and negatively co-expressed with IGFBP3/5/7 based on TCGA data and constructed a heatmap (**Figures 4A–C**). The thresholds for gene co-expression genes were |r| > 0.4 and p < 0.001. We found 449 genes co-expressed with IGFBP3, 2,295 with IGFBP5, and 2,643 with IGFBP7; 407 genes overlapped the three co-expression groups (**Figure 4D**, all genes listed in **Supplementary Table 2**). GO (**Figure 4E**) and KEGG (**Figure 4F**) analyses were performed for all four groups of genes with R package “clusterProfiler” (**Supplementary Table 3**). Collagen-containing extracellular matrix (ECM), extracellular structure organization, ECM organization, and ECM structural constituent were the most significant terms in the GO analysis. In the KEGG analysis, ECM–receptor interaction, focal adhesion, and PI3K-Akt signaling pathway were the most significant pathways. These results suggest that IGFBP3/5/7 might be involved in tumor progression *via* interactions with the ECM. Besides, the association between tumor stage and IGFBP expression was also validated in the other two cohorts (20, 22) (**Supplementary Figure 2**).

### Genetic Alterations of IGFBP1–7 in STAD Patients

**Figure 5:**
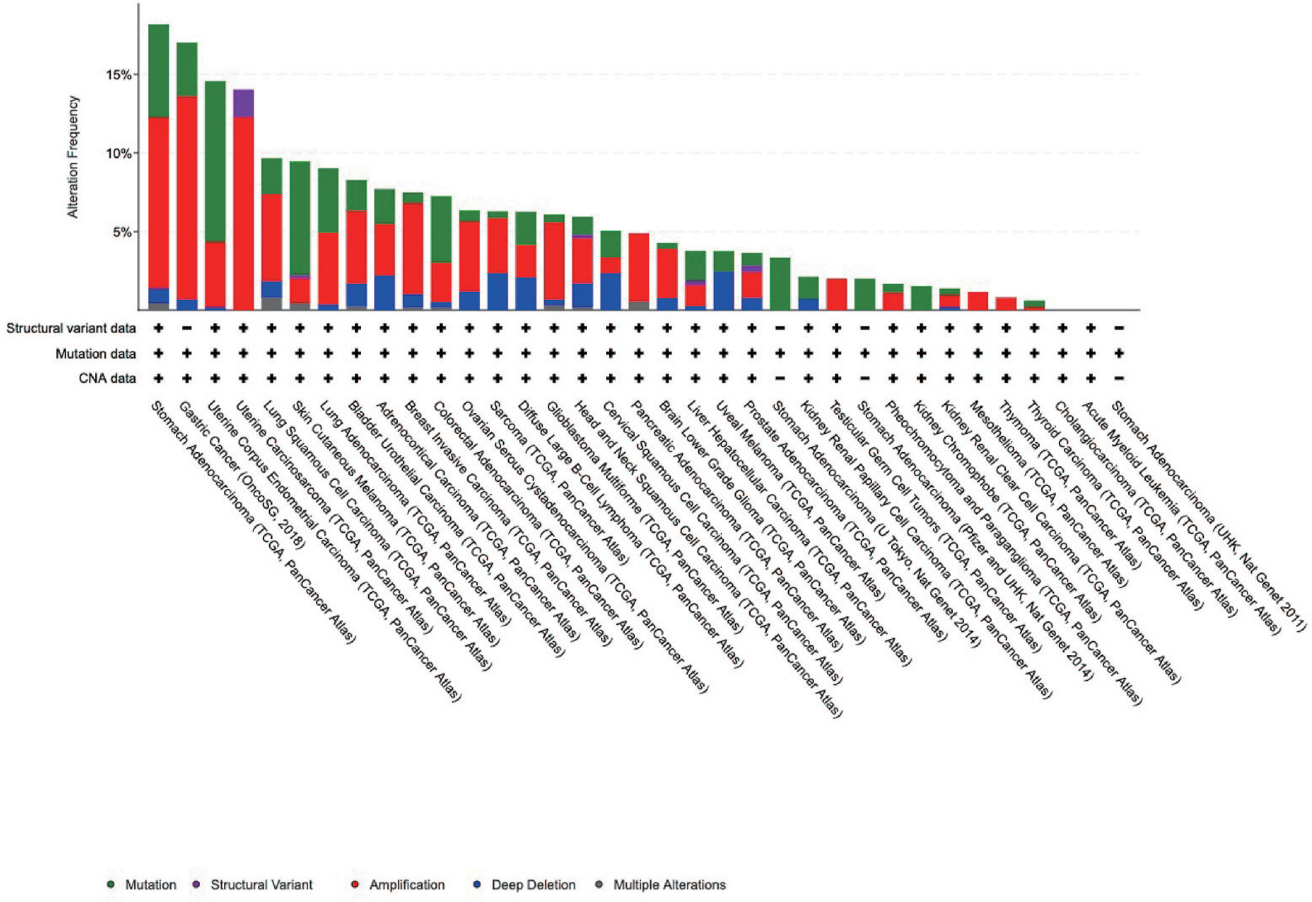
Genetic alterations of IGFBP1–7 in different cancer patients from cBioPortal database.

Pathogenic mutations increase the risk of tumorigenesis, including that of gastric cancer. We analyzed the genetic alterations of IGFBP1–7 in STAD patients using cBioPortal; 35 datasets and 11,070 patients were included in this analysis. The results showed that STAD patients had the highest rates of IGFBP1–7 genetic alterations compared with other cancer types, with 80 of 440 patients (18.8%) having such alterations according to TCGA data. In the OncoSG (2018) database, 25 of 147 gastric cancer patients (17.1%) had such genetic mutations (**Figure 5**). These results further confirmed the importance of IGFBP1–7 in gastric cancer (specific mutation types are listed in **Supplementary Table 4**). However, mutations were not associated with prognosis in STAD patients (**Supplementary Figure 3**).

### Interactions of IGFBPs in STAD and Hub Hene Analyses

**Figure 6:**
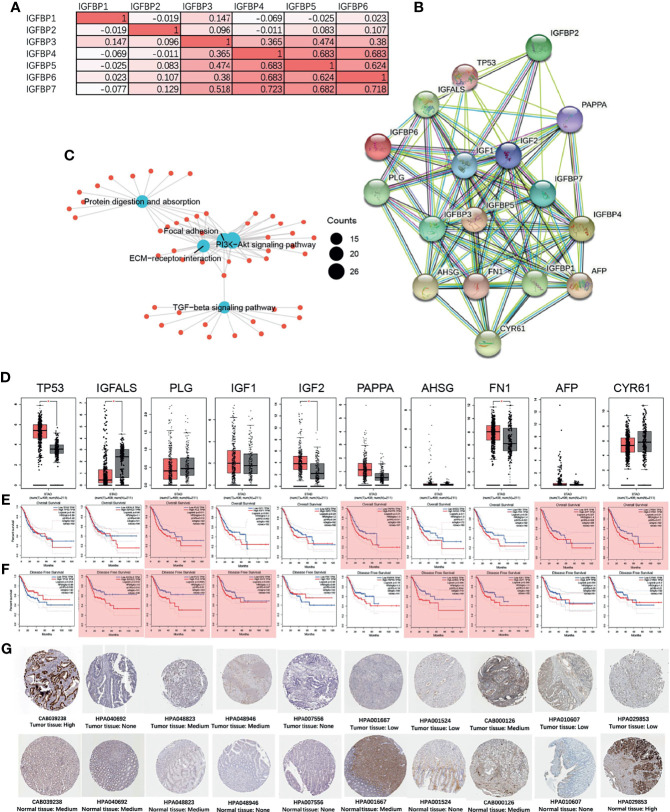
IGFBP1–7 gene expression correlation and protein network interactions (STRING), and hub gene expression and survival analysis of STAD based on GEPIA database. **(A)** Correlations among IGFBP1–7 in STAD patients from the TCGA database. **(B)** PPI network of IGFBP1–7. **(C)** GO analysis of interacting proteins from **(B)**. **(D)** Expression levels of 10 hub genes of IGFBP1–7 in STAD patients. **(E)** Overall survival analysis for the 10 hub genes in GEPIA database. **(F)** Disease-free survival analysis for the 10 hub genes in the GEPIA database. *p < 0.05 (bar plot); p < 0.05 marked as red shadow (survival analysis). **(G)** The immunohistochemical verification of IGFBP1–7 in patients’ tissue from Human Protein Atlas data (antibody name and tissue type are listed below the immunohistochemical figure).

Next, we performed a correlation analysis for IGFBP1–7 and found that IGFBP3/4/5/6/7 had strong correlations with each other (**Figure 6A**). Then, we put all seven molecules into STRING and constructed a PPI network (**Figure 6B**, the interaction score was >0.4) with IGFBP1/3/4/5/7 in the center and another 10 hub genes (TP53, IGFALS, PLG, IGF1, IGF2, PAPPA, AHSG, FN1, AFP, and CYR61) around them. We performed GO analysis for all these genes and found that they encoded proteins involved in the PI3K-Akt signaling pathway and ECM–reception interaction (**Figure 6C**). Expression level and survival analyses were also performed on these hub genes (**Figure 6D**). TP53 and IGF2 expression were significantly elevated in STAD patients, but they were not associated with overall (**Figure 6E**) or disease-free survival (**Figure 6F**). PLG, PAPPA, AFP, and CYR61 were associated with overall survival, whereas IGFALS, PLG, IGF1, AHSG, and FN1 were associated with disease-free survival. The immunohistochemical verification from Human Protein Atlas data is shown in **Figure 6G**.

### Association of Immune Cell Infiltration With IGFBP1–7 in STAD Patients

**Figure 7:**
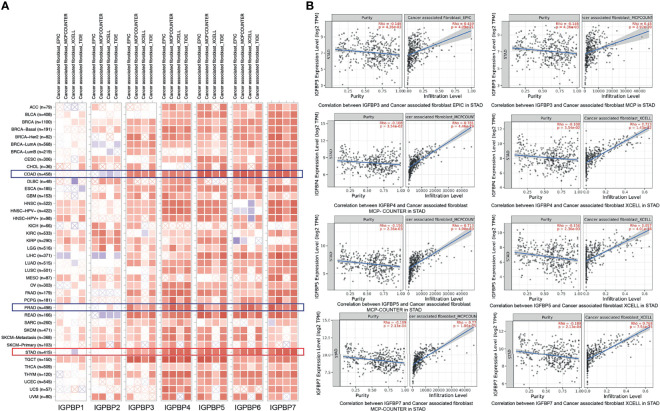
Correlations between differentially expressed IGFBPs and immune cell infiltration (TIMER). **(A)** Correlations between abundance of immune cells and expression of IGFBP1–7 in 40 different cancer types. **(B)** Examples of IGFBP3/4/5/7-related tumor immune cell infiltration in STAD patients.

Immune cell infiltration creates a microenvironment for the tumor that facilitates cancer cell proliferation and progression. The relationships between IGFBP1–7 expression and immune cell infiltration were analyzed using the TIMER database (18, 23). Using the EPIC, MCPCOUNTER, XCELL, and TIDE algorithms, we found that IGFBP3/4/5/6/7 were all associated with cancer-associated fibroblast (CAF) infiltration in STAD, COAD (colon adenocarcinoma), and READ (rectal adenocarcinoma) (**Figure 7A**). **Figure 7B** shows some examples of specific correlations of IGFBP with CAFs in STAD. These results further indicate that IGFBP3–7 have important roles in the TME. The correlation of IGFBP expression and other subtypes of immune cell infiltration including B cells, CD4+T cells, CD8+T cells, neutrophils, macrophages, and dendritic cells in patients with gastric cancer is shown in **Supplementary Figure 4**.

## Discussion

Gastric cancer is the third most common cause of cancer-related deaths worldwide, with a particularly high incidence in Asian populations. Many studies have been devoted to investigating the pathogenesis of gastric cancer and identifying prognostic biomarkers. Among such markers, IGFBPs have been shown to modulate cell proliferation, migration, and autophagy *via* temporal and spatial regulation of IGF and IGFR levels (24). However, their roles in the occurrence and development of gastric cancer remained controversial. The results of this study showed that IGFBP1/3/7 expression levels in STAD tissue were significantly higher than those in normal tissues. IGFBP1/5/7 expression was significantly associated with overall survival, whereas IGFBP6/7 expression was significantly correlated with disease-free survival. IGFBP3/5/6/7 expression was significantly elevated in pathological stages II–IV compared with pathological stage I. IGFBP3/5/7 expression was also significantly increased in advanced T-stage patients and was associated with tumor progression in STAD. Collagen-containing ECM, extracellular structure organization, ECM organization, and ECM structural constituents were the main GO/KEGG terms correlated with IGFBP3/5/7 genes. STAD and gastric cancer had the most IGFBP1–7 mutations compared with other tumors. In the hub gene analysis, expression levels of TP53 and IGF2 were significantly elevated in STAD patients; PLG, PAPPA, AFP, and CYR61 were associated with overall survival rate; and IGFALS, PLG, IGF1, AHSG, and FN1 were associated with disease-free survival rate. Finally, IGFBP3-7 expression levels were all correlated with CAF infiltration in STAD, COAD, and READ.

IGFBPs show variable expressions in gastric cancer tissues and cell lines, and there has been no comprehensive evaluation of IGFBPs as biomarkers in gastric cancer. A study of 11 gastric cancer cell lines demonstrated that IGFBP1 expression levels were extremely low in all cell lines, whereas IGFBP2 and IGFBP4 were expressed in 10 and 9 cell lines, respectively, and IGFBP3, IGFBP5, and IGFBP6 were expressed in half of all cell lines (25). Among these IGFBPs, IGFBP3, and IGFBP5 have received more research focus than others. Our data showed higher IGFBP3 expression levels in STAD patients’ tumor tissues but no relationship with OS. Other studies found that serum IGFBP3 levels were similar between cancer and control groups, but surgery could reduce serum IGFBP3 levels by decreasing IGFBP3 protease activity (26). Another study examined tumor tissues and adjacent tumor-free tissues from 86 STAD patients; the results showed that IGFBP3 expression was higher in the tumor-free tissues, and high IGFBP3 expression predicted better prognosis (27). All these studies illustrate the complex relationship between IGFBP3 and gastric cancer. Studies of other tumor types have provided some insight into the specific mechanisms of IGFBP3. For example, cancer-related gene vasohibin-2 induced proliferation of breast cancer cells by activating IGFBP3 and IGFBP6 (28).

This study first proposed that IGFBP7 might affect gastric cancer development by modulating the ECM. IGFBP7 is upregulated in gastric cancer and located in the cytoplasm of the majority of cancer cells, fibroblasts, and lymphocytes, and its expression is significantly correlated with indicators of pathological stage including tumor invasion depth, lymph node metastasis, and distant metastasis/recurrence (29). Regarding pathological typing, IGFBP7 has been shown to be upregulated in undifferentiated compared with differentiated tumors (13). The cell matrix is widely understood to be involved in cancer occurrence, progression, and metabolism (30). Disruption of the normal structure and function of gastric epithelia eventually leads to gastric cancer progression. However, few studies have investigated the relationship between IGFBP7 and collagen-containing ECM formation in gastric cancer. A study compared premalignant and malignant stomach lesions and found that collagen-related genes COL11A1 and COL1A1 involved the focal adhesion pathway (31). In our study, COL1A1 was found to be co-expressed with IGFBP7 in STAD patients with |r| = 0.55355494 and p = 1.71475E-31. COL4A1 overexpression has previously been shown to be correlated with overall survival in gastric cancer (32); it was correlated with IGFBP7 with p = 6.99147E-33 in our study. Another overexpressed collagen gene, COL6A3 (33), was correlated with IGFBP7 with p = 1.48823E-40. COL12A1 was upregulated in gastric cancer and positively associated with tumor invasion and clinical stage and was also significantly correlated with IGFBP7 (|r| = 0.426016684, p = 5.75765E-18). Although collagen and IGFBP7 were all closely related to cancer progression, few studies have focused on the contribution of IGFBP7 in gastric cancer. However, research from other perspectives has demonstrated a relationship between IGFBP7 and collagen. Human endometrium cells formed a mesh-like structure in human uterus as well as on Matrigel *in vitro*. Knockdown of IGFBP7 could inhibit the formation of this mesh-like structure by interfering with protein kinase A and the MAPK signaling pathway (34). In a wound healing study, wound healing mediators including TGF-β1 and chemokines IL-6, IL-8, MCP-1, and RANTES in mesenchymal stem cells were identified, as well as IGFBP7, indicating that IGFBP7 contributes to the formation of the ECM (35).

In recent years, the TME has become a research hot spot. Accumulating evidence shows that carcinomas modify their environment by expressing growth factors, altering ECM gene expression to increase fibroblast proliferation, and changing immune cell infiltration, as well as by cross-talking with each other (36). During this process, growth factors and CAFs play important parts (37). IGFBP7 was identified as a fibroblast marker in CAFs and significantly stimulated fibroblast proliferation and migration (38). In gastric cancer, the abnormal expression of FGF9 in lymph node CAFs was correlated with poor prognosis (39). *H. pylori* infection was shown to elevate VCAM1 expression in CAFs, which indicated tumor invasion and progression (40). IGFBPs facilitate binding of IGF1 to ECM prote*in vitro*nectin to stimulate proliferation and migration of skin keratinocytes and fibroblasts (12). IGFs independently stimulate IGFBP3 and reduce IGFBP4 in human fibroblasts and epidermal cells (41). Knockout of Igfbp7 increased the proliferation of mouse hepatocytes and embryonic fibroblasts, whereas its overexpression inhibited hepatocytes in syngeneic immunocompetent mice, indicating its immune-mediated function (42). However, there has been a lack of studies focusing on IGFBPs in CAFs in gastric cancer, although these molecules have been shown to have an important role in fibroblasts. The mechanism by which IGFBPs participate in gastric cancer progression and metastasis *via* CAFs is worth further exploration. In addition, stromal cells are much more stable than cancer cells, which makes them attractive therapeutic targets for gastric cancer treatment (37, 43).

This study had some limitations. All data were downloaded from online databases and analyzed by computer algorithms; further studies including cell and animal experiments are required to validate the results. However, the mechanism of IGFBPs’ involvement in tumor progression could become a new research direction and provide promising treatment targets.

## Conclusion

In conclusion, we systematically analyzed the transport protein IGFBP1–7 in gastric cancer. With collection of the gene expression data of cancer *vs.* normal patients, tumor *vs.* adjacent tumor tissue, and IGFBP mutations in all cancer types and immune infiltration data, we provided a relative complete analysis for IGFBP1–7 in gastric cancer. Our results screened out the meaningful IGFBPs in gastric cancer clinical prognosis, tumor staging, and immune infiltration and provided directions for the future research on gastric cancer. To better elucidate how these molecules get involved in specific mechanisms of gastric cancer occurrence, progression, and metastasis, further efforts might be focused on the research of IGFBPs in the tumor microenvironment and extracellular matrix.

## Data Availability Statement

The datasets presented in this study can be found in online repositories. The names of the repository/repositories and accession number(s) can be found in the article/**Supplementary Material**.

## Author Contributions

QL and YF designed the study and performed the bioinformatics analysis. QL and MXZ collected TCGA data and participated in the writing of the manuscript and did the main revision. All authors contributed to manuscript revision and read and approved the submitted version.

## Funding

This project was supported by the National Natural Science Foundation of China (No. 8210061367) for MXZ, (No. 81871995) for YF, Henan Health Department (No. YXKC2020029) and the Excellent Youth Fund of Henan Natural Science Foundation (No. 212300410075) for YF, Henan Province Medical Science and Technology Co-construction Project for QL, JWJ and MXZ.

## Conflict of Interest

The authors declare that the research was conducted in the absence of any commercial or financial relationships that could be construed as a potential conflict of interest.

The reviewer FM declared a shared affiliation with the authors to the handling editor at the time of the review.

## Publisher’s Note

All claims expressed in this article are solely those of the authors and do not necessarily represent those of their affiliated organizations, or those of the publisher, the editors and the reviewers. Any product that may be evaluated in this article, or claim that may be made by its manufacturer, is not guaranteed or endorsed by the publisher.

## Supplementary Material

Supplementary Figure 1 Validation of survival analysis of IGFBP1, IGFBP3, IGFBP7 in STAD patients from Chen’s data [PMID: 12925757]. There was no significant difference in IGFBP1/3/7.

Supplementary Figure 2 Validation of IGPBP1–7 expression and tumor stage in STAD patients from Cho’s data and Chen’s data. **(A)** The expression difference of IGFBP1-7 between T stage II and T stage III from Cho’s data [PMID: 19798449]. **(B)** The expression difference of ten hub genes between T stage II and T stage III from Cho’s data. **(C)** The expression difference of IGFBP1, IGFBP3, IGFBP7, TP53, PLG, IGF2, PAPPA, AHSG, FN1, AFP, CYR1 between T stage I, II, III, IV from Chen’s data [PMID: 12925757].

Supplementary Figure 3 Survival curves of IGFBP associated mutations in STAD patients.

Supplementary Figure 4 Correlation of IGFBPs expression immune cell infiltration including B cells, CD4+ cells, CD8+ cells, macrophages, neutrophils and dendritic cells in gastric cancer.

Supplementary Table 1 Cox multivariate regression analysis for IGFBP1/3/5/7 in STAD patients with TCGA database.

Supplementary Table 2 IGFBP3/5/7 co-expressed genes and overlapping genes of three co-expression groups.

Supplementary Table 3 GO and KEGG analysis of IGFBP3/5/7 co-expressed genes.

Supplementary Table 4 Specific mutation sites of genetic alterations of IGFBP1–7 in STAD patients.

## References

[1] Smyth EC Nilsson M Grabsch HI van Grieken NC Lordick F. Gastric Cancer. Lancet (2020) 396:635–48. doi: 10.1016/S0140-6736(20)31288-5 32861308

[2] McGuire S. World Cancer Report 2014. Geneva, Switzerland: World Health Organization, International Agency for Research on Cancer, WHO Press, 2015. Adv Nutr (2016) 7:418–9. doi: 10.3945/an.116.012211 PMC4785485 26980827

[3] Bray F Ferlay J Soerjomataram I Siegel RL Torre LA Jemal A. Global Cancer Statistics 2018: GLOBOCAN Estimates of Incidence and Mortality Worldwide for 36 Cancers in 185 Countries. CA Cancer J Clin (2018) 68:394–424. doi: 10.3322/caac.21492 30207593

[4] Quezada-Marin JI Lam AK Ochiai A Odze RD Washington KM Fukayama M. Gastrointestinal Tissue-Based Molecular Biomarkers: A Practical Categorisation Based on the 2019 World Health Organization Classification of Epithelial Digestive Tumours. Histopathology (2020) 77:340–50. doi: 10.1111/his.14120 32320495

[5] Oliveira C Pinheiro H Figueiredo J Seruca R Carneiro F. Familial Gastric Cancer: Genetic Susceptibility, Pathology, and Implications for Management. Lancet Oncol (2015) 16:e60–70. doi: 10.1016/S1470-2045(14)71016-2 25638682

[6] van der Post RS Vogelaar IP Carneiro F Guilford P Huntsman D Hoogerbrugge N. Hereditary Diffuse Gastric Cancer: Updated Clinical Guidelines With an Emphasis on Germline CDH1 Mutation Carriers. J Med Genet (2015) 52:361–74. doi: 10.1136/jmedgenet-2015-103094 PMC4453626 25979631

[7] Donner I Kiviluoto T Ristimaki A Aaltonen LA Vahteristo P. Exome Sequencing Reveals Three Novel Candidate Predisposition Genes for Diffuse Gastric Cancer. Fam Cancer (2015) 14:241–6. doi: 10.1007/s10689-015-9778-z 25576241

[8] Fewings E Larionov A Redman J Goldgraben MA Scarth J Richardson S. Germline Pathogenic Variants in PALB2 and Other Cancer-Predisposing Genes in Families With Hereditary Diffuse Gastric Cancer Without CDH1 Mutation: A Whole-Exome Sequencing Study. Lancet Gastroenterol Hepatol (2018) 3:489–98. doi: 10.1016/S2468-1253(18)30079-7 PMC5992580 29706558

[9] Li J Woods SL Healey S Beesley J Chen X Lee JS. Point Mutations in Exon 1B of APC Reveal Gastric Adenocarcinoma and Proximal Polyposis of the Stomach as a Familial Adenomatous Polyposis Variant. Am J Hum Genet (2016) 98:830–42. doi: 10.1016/j.ajhg.2016.03.001 PMC4863475 27087319

[10] Tsugane S Sasazuki S. Diet and the Risk of Gastric Cancer: Review of Epidemiological Evidence. Gastric Cancer (2007) 10:75–83. doi: 10.1007/s10120-007-0420-0 17577615

[11] Kashyap MK. Role of Insulin-Like Growth Factor-Binding Proteins in the Pathophysiology and Tumorigenesis of Gastroesophageal Cancers. Tumour Biol (2015) 36:8247–57. doi: 10.1007/s13277-015-3972-3 26369544

[12] Murekatete B Shokoohmand A McGovern J Mohanty L Meinert C Hollier BG. Targeting Insulin-Like Growth Factor-I and Extracellular Matrix Interactions in Melanoma Progression. Sci Rep (2018) 8:583. doi: 10.1038/s41598-017-19073-4 29330502 PMC5766529

[13] Takeno A Takemasa I Doki Y Yamasaki M Miyata H Takiguchi S. Integrative Approach for Differentially Overexpressed Genes in Gastric Cancer by Combining Large-Scale Gene Expression Profiling and Network Analysis. Br J Cancer (2008) 99:1307–15. doi: 10.1038/sj.bjc.6604682 PMC2570518 18827816

[14] Rhodes DR Yu J Shanker K Deshpande N Varambally R Ghosh D. ONCOMINE: A Cancer Microarray Database and Integrated Data-Mining Platform. Neoplasia (2004) 6:1–6. doi: 10.1016/S1476-5586(04)80047-2 15068665 PMC1635162

[15] Tang Z Li C Kang B Gao G Li C Zhang Z. GEPIA: A Web Server for Cancer and Normal Gene Expression Profiling and Interactive Analyses. Nucleic Acids Res (2017) 45:W98–102. doi: 10.1093/nar/gkx247 28407145 PMC5570223

[16] Gao J Aksoy BA Dogrusoz U Dresdner G Gross B Sumer SO. Integrative Analysis of Complex Cancer Genomics and Clinical Profiles Using the Cbioportal. Sci Signal (2013) 6:pl1. doi: 10.1126/scisignal.2004088 23550210 PMC4160307

[17] Szklarczyk D Gable AL Lyon D Junge A Wyder S Huerta-Cepas J. STRING V11: Protein-Protein Association Networks With Increased Coverage, Supporting Functional Discovery in Genome-Wide Experimental Datasets. Nucleic Acids Res (2019) 47:D607–13. doi: 10.1093/nar/gky1131 PMC6323986 30476243

[18] Li T Fan J Wang B Traugh N Chen Q Liu JS. TIMER: A Web Server for Comprehensive Analysis of Tumor-Infiltrating Immune Cells. Cancer Res (2017) 77:e108–10. doi: 10.1158/0008-5472.CAN-17-0307 PMC6042652 29092952

[19] Yu G Wang LG Han Y He QY. Clusterprofiler: An R Package for Comparing Biological Themes Among Gene Clusters. OMICS (2012) 16:284–7. doi: 10.1089/omi.2011.0118 PMC3339379 22455463

[20] Chen X Leung SY Yuen ST Chu KM Ji J Li R. Variation in Gene Expression Patterns in Human Gastric Cancers. Mol Biol Cell (2003) 14:3208–15. doi: 10.1091/mbc.e02-12-0833 PMC181561 12925757

[21] Wang Q Wen YG Li DP Xia J Zhou CZ Yan DW. Upregulated INHBA Expression is Associated With Poor Survival in Gastric Cancer. Med Oncol (2012) 29:77–83. doi: 10.1007/s12032-010-9766-y 21132402

[22] Ooi CH Ivanova T Wu J Lee M Tan IB Tao J. Oncogenic Pathway Combinations Predict Clinical Prognosis in Gastric Cancer. PloS Genet (2009) 5:e1000676. doi: 10.1371/journal.pgen.1000676 19798449 PMC2748685

[23] Li T Fu J Zeng Z Cohen D Li J Chen Q. TIMER2.0 for Analysis of Tumor-Infiltrating Immune Cells. Nucleic Acids Res (2020) 48:W509–14. doi: 10.1093/nar/gkaa407 PMC7319575 32442275

[24] Bach LA. IGF-Binding Proteins. J Mol Endocrinol (2018) 61:T11–28. doi: 10.1530/JME-17-0254 29255001

[25] Yi HK Hwang PH Yang DH Kang CW Lee DY. Expression of the Insulin-Like Growth Factors (IGFs) and the IGF-Binding Proteins (IGFBPs) in Human Gastric Cancer Cells. Eur J Cancer (2001) 37:2257–63. doi: 10.1016/S0959-8049(01)00269-6 11677116

[26] Lee DY Yang DH Kang CW Kim SJ Joo CU Cho SC. Serum Insulin-Like Growth Factors (IGFs) and IGF Binding Protein (IGFBP)-3 in Patients With Gastric Cancer: IGFBP-3 Protease Activity Induced by Surgery. J Korean Med Sci (1997) 12:32–9. doi: 10.3346/jkms.1997.12.1.32 PMC3054268 9142657

[27] Xue M Fang Y Sun G Zhuo W Zhong J Qian C. IGFBP3, a Transcriptional Target of Homeobox D10, is Correlated With the Prognosis of Gastric Cancer. PloS One (2013) 8:e81423. doi: 10.1371/journal.pone.0081423 24386080 PMC3873913

[28] Tu M Liu X Han B Ge Q Li Z Lu Z. Vasohibin2 Promotes Proliferation in Human Breast Cancer Cells *via* Upregulation of Fibroblast Growth Factor2 and Growth/Differentiation Factor15 Expression. Mol Med Rep (2014) 10:663–9. doi: 10.3892/mmr.2014.2317 PMC4094825 24920244

[29] Sato Y Inokuchi M Takagi Y Otsuki S Fujimori Y Yanaka Y. Relationship Between Expression of IGFBP7 and Clinicopathological Variables in Gastric Cancer. J Clin Pathol (2015) 68:795–801. doi: 10.1136/jclinpath-2015-202987 26043748

[30] Moreira AM Pereira J Melo S Fernandes MS Carneiro P Seruca R. The Extracellular Matrix: An Accomplice in Gastric Cancer Development and Progression. Cells (2020) 9:394. doi: 10.3390/cells9020394 PMC7072625 32046329

[31] Zhao Y Zhou T Li A Yao H He F Wang L. A Potential Role of Collagens Expression in Distinguishing Between Premalignant and Malignant Lesions in Stomach. Anat Rec (Hoboken) (2009) 292:692–700. doi: 10.1002/ar.20874 19306436

[32] Zhang QN Zhu HL Xia MT Liao J Huang XT Xiao JW. A Panel of Collagen Genes Are Associated With Prognosis of Patients With Gastric Cancer and Regulated by microRNA-29c-3p: An Integrated Bioinformatics Analysis and Experimental Validation. Cancer Manag Res (2019) 11:4757–72. doi: 10.2147/CMAR.S198331 PMC6538884 31213898

[33] Xie X Liu X Zhang Q Yu J. Overexpression of Collagen VI Alpha3 in Gastric Cancer. Oncol Lett (2014) 7:1537–43. doi: 10.3892/ol.2014.1910 PMC3997710 24765172

[34] Kutsukake M Tamura K Yoshie M Tachikawa E. Knockdown of IGF-Binding Protein 7 Inhibits Transformation of the Endometrial Gland in an *In Vitro* Model. Mol Reprod Dev (2010) 77:265–72. doi: 10.1002/mrd.21143 20029996

[35] Walter MN Wright KT Fuller HR MacNeil S Johnson WE. Mesenchymal Stem Cell-Conditioned Medium Accelerates Skin Wound Healing: An *In Vitro* Study of Fibroblast and Keratinocyte Scratch Assays. Exp Cell Res (2010) 316:1271–81. doi: 10.1016/j.yexcr.2010.02.026 20206158

[36] Quail DF Joyce JA. Microenvironmental Regulation of Tumor Progression and Metastasis. Nat Med (2013) 19:1423–37. doi: 10.1038/nm.3394 PMC3954707 24202395

[37] Bhowmick NA Neilson EG Moses HL. Stromal Fibroblasts in Cancer Initiation and Progression. Nature (2004) 432:332–7. doi: 10.1038/nature03096 PMC3050735 15549095

[38] Komiya E Furuya M Watanabe N Miyagi Y Higashi S Miyazaki K. Elevated Expression of Angiomodulin (AGM/IGFBP-Rp1) in Tumor Stroma and its Roles in Fibroblast Activation. Cancer Sci (2012) 103:691–9. doi: 10.1111/j.1349-7006.2012.02203.x PMC7659301 22321149

[39] Wang R Sun Y Yu W Yan Y Qiao M Jiang R. Downregulation of miRNA-214 in Cancer-Associated Fibroblasts Contributes to Migration and Invasion of Gastric Cancer Cells Through Targeting FGF9 and Inducing EMT. J Exp Clin Cancer Res (2019) 38:20. doi: 10.1186/s13046-018-0995-9 30646925 PMC6334467

[40] Shen J Zhai J You Q Zhang G He M Yao X. Cancer-Associated Fibroblasts-Derived VCAM1 Induced by H. Pylori Infection Facilitates Tumor Invasion in Gastric Cancer. Oncogene (2020) 39:2961–74. doi: 10.1038/s41388-020-1197-4 32034307

[41] Neely EK Rosenfeld RG. Insulin-Like Growth Factors (IGFs) Reduce IGF-Binding Protein-4 (IGFBP-4) Concentration and Stimulate IGFBP-3 Independently of IGF Receptors in Human Fibroblasts and Epidermal Cells. Endocrinology (1992) 130:985–93. doi: 10.1210/endo.130.2.1370799 1370799

[42] Akiel M Guo C Li X Rajasekaran D Mendoza RG Robertson CL. IGFBP7 Deletion Promotes Hepatocellular Carcinoma. Cancer Res (2017) 77:4014–25. doi: 10.1158/0008-5472.CAN-16-2885 PMC5894280 28619711

[43] Kalluri R. The Biology and Function of Fibroblasts in Cancer. Nat Rev Cancer (2016) 16:582–98. doi: 10.1038/nrc.2016.73 27550820

