# Supplementary figures and images for: Comprehensive Analysis of IGFBPs as Biomarkers in Gastric Cancer
Source: Front Oncol. 2021 Oct 21;11:723131. doi: 10.3389/fonc.2021.723131 (PMC8567138; doi:10.3389/fonc.2021.723131)

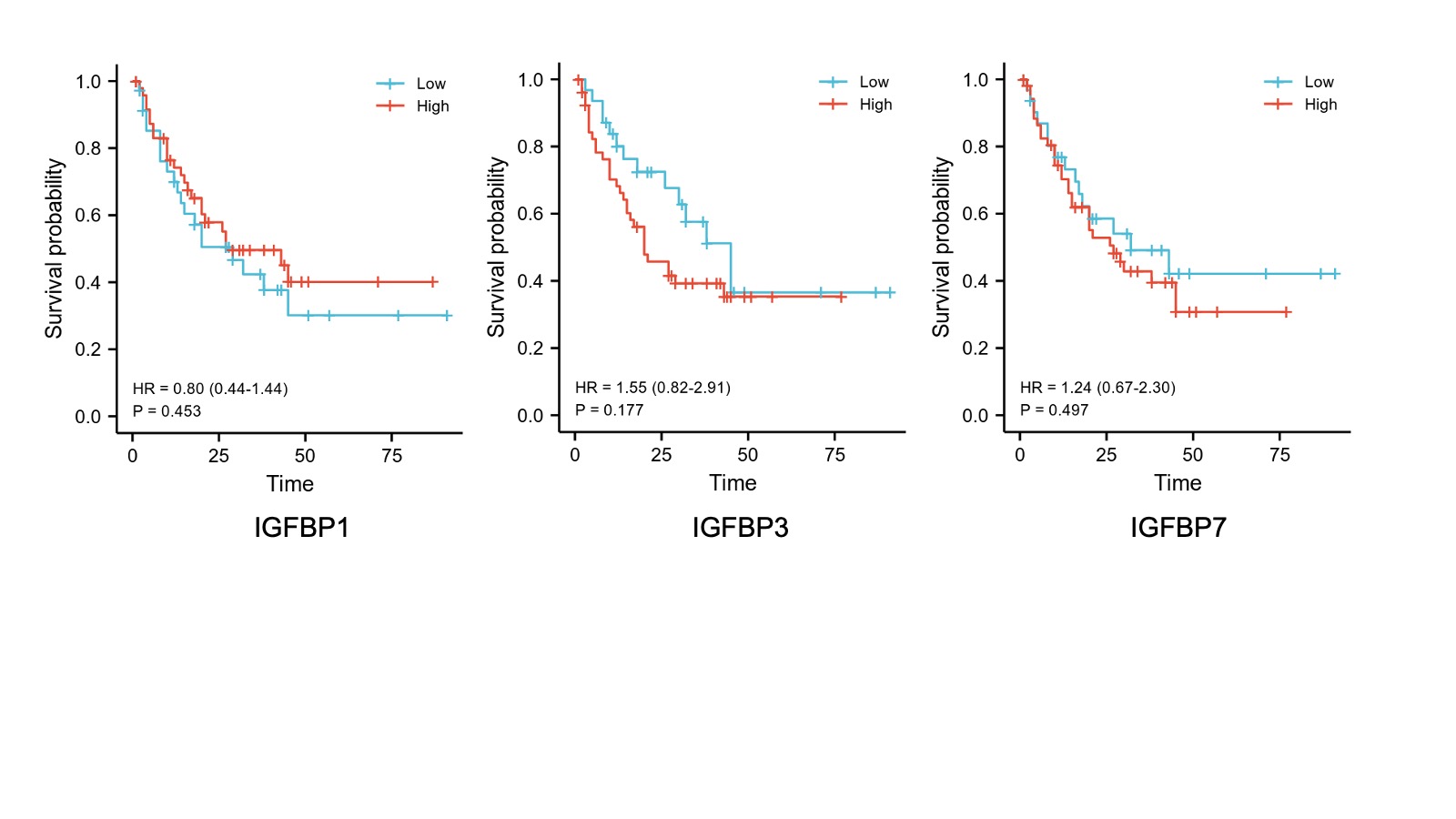

Supplement: Supplementary Figure 1 — Validation of survival analysis of IGFBP1, IGFBP3, IGFBP7 in STAD patients from Chen’s data [PMID: 12925757]. There was no significant difference in IGFBP1/3/7. [file Image_1.jpeg]

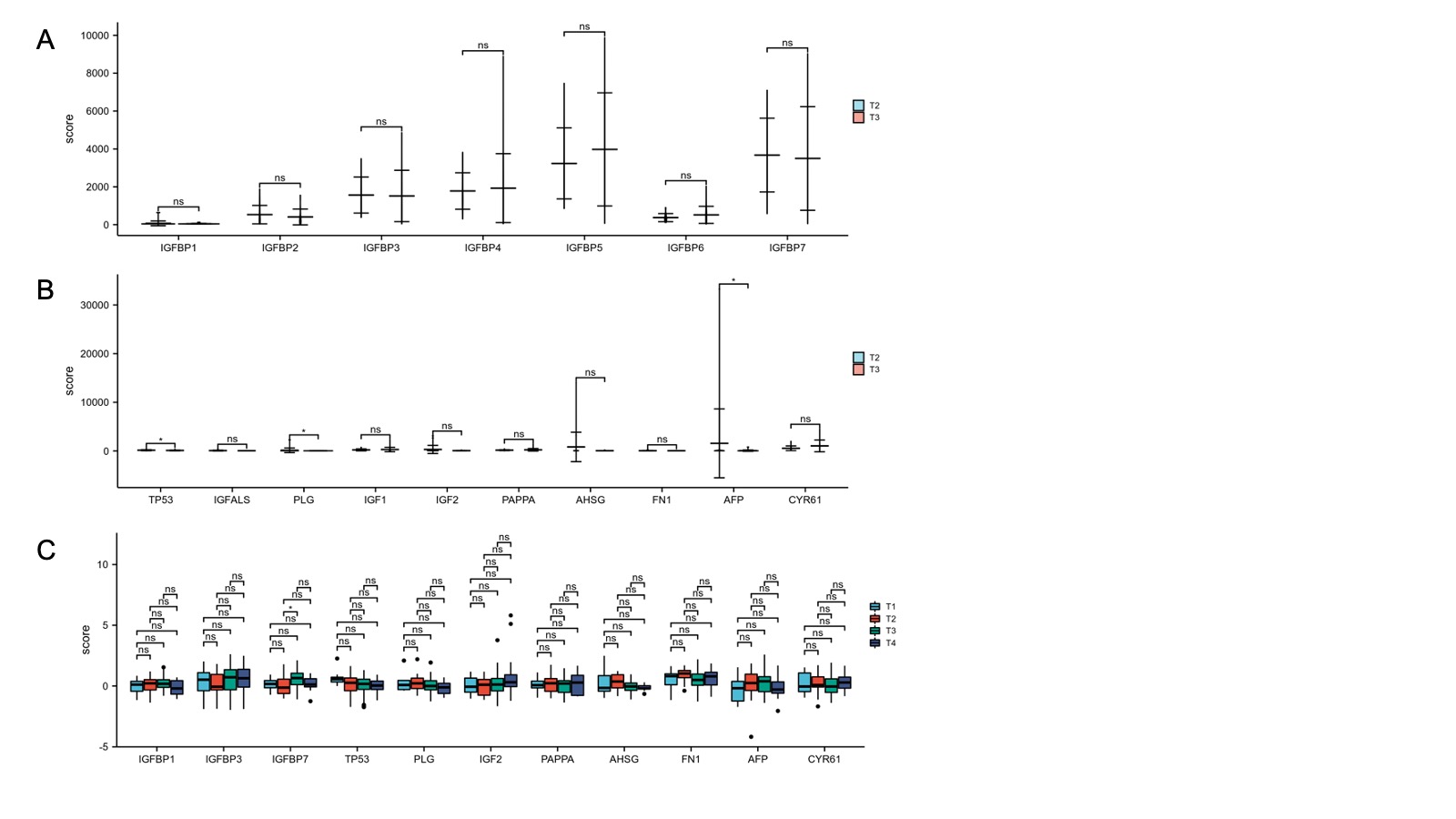

Supplement: Supplementary Figure 2 — Validation of IGPBP1–7 expression and tumor stage in STAD patients from Cho’s data and Chen’s data. (A) The expression difference of IGFBP1-7 between T stage II and T stage III from Cho’s data [PMID: 19798449]. (B) The expression difference of ten hub genes between T stage II and T stage III from Cho’s data. (C) The expression difference of IGFBP1, IGFBP3, IGFBP7, TP53, PLG, IGF2, PAPPA, AHSG, FN1, AFP, CYR1 between T stage I, II, III, IV from Chen’s data [PMID: 12925757]. [file Image_2.jpeg]

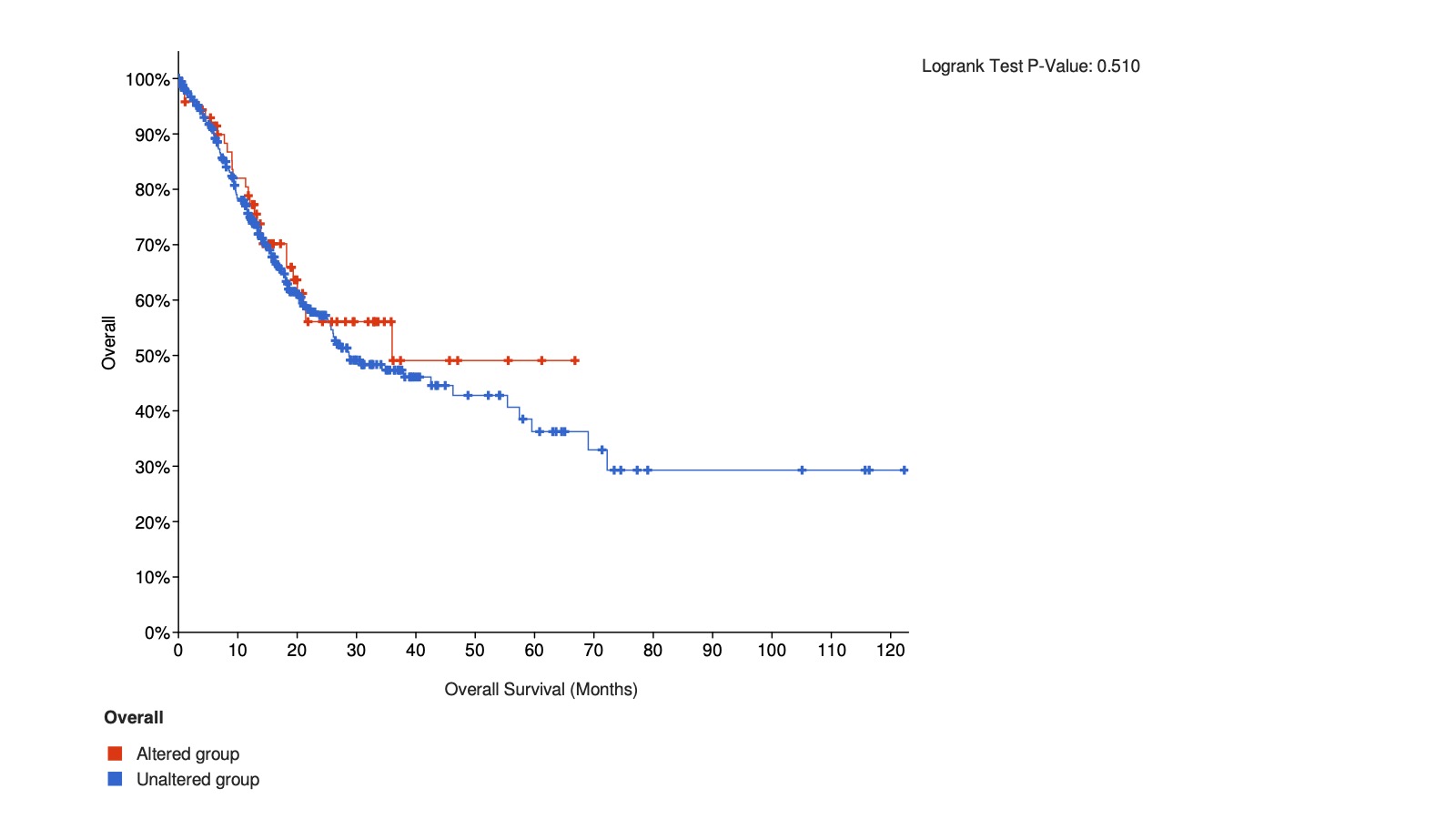

Supplement: Supplementary Figure 3 — Survival curves of IGFBP associated mutations in STAD patients. [file Image_3.jpeg]

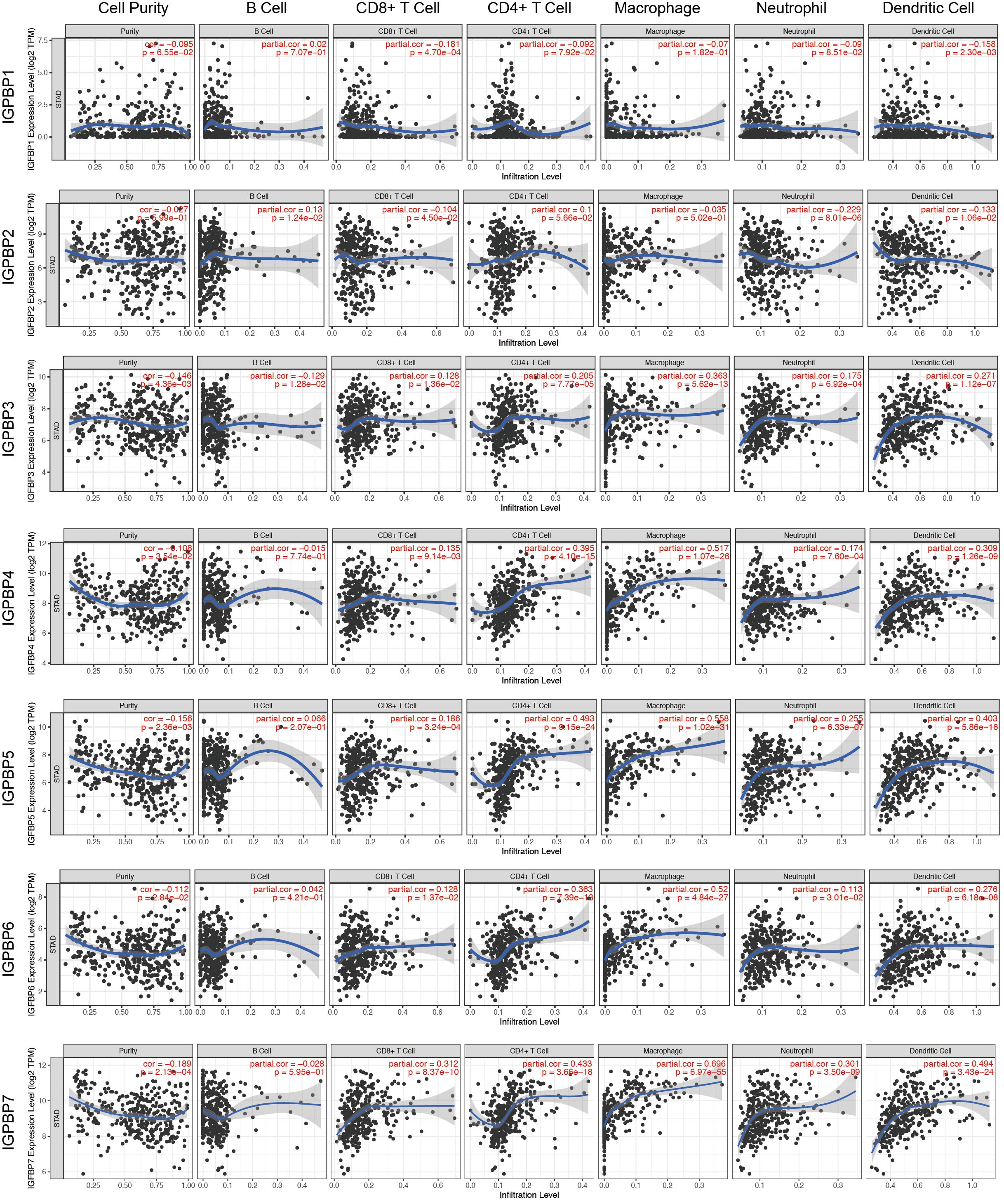

Supplement: Supplementary Figure 4 — Correlation of IGFBPs expression immune cell infiltration including B cells, CD4+ cells, CD8+ cells, macrophages, neutrophils and dendritic cells in gastric cancer. [file Image_4.jpeg]
